# Cell Therapy in Patients with Critical Limb Ischemia

**DOI:** 10.1155/2015/931420

**Published:** 2015-08-02

**Authors:** Rita Compagna, Bruno Amato, Salvatore Massa, Maurizio Amato, Raffaele Grande, Lucia Butrico, Stefano de Franciscis, Raffaele Serra

**Affiliations:** ^1^Interuniversity Center of Phlebolymphology (CIFL), International Research and Educational Program in Clinical and Experimental Biotechnology, Headquarters, University Magna Graecia of Catanzaro, Viale Europa, 88100 Catanzaro, Italy; ^2^Department of Clinical Medicine and Surgery, University of Naples “Federico II”, 80100 Naples, Italy; ^3^Department of General, Geriatric, Oncologic Surgery and Advanced Technologies, University of Naples “Federico II”, 80100 Naples, Italy; ^4^Department of Medical and Surgical Sciences, University of Catanzaro, 88100 Catanzaro, Italy

## Abstract

Critical limb ischemia (CLI) represents the most advanced stage of peripheral arterial obstructive disease (PAOD) with a severe obstruction of the arteries which markedly reduces blood flow to the extremities and has progressed to the point of severe rest pain and/or even tissue loss. Recent therapeutic strategies have focused on restoring this balance in favor of tissue survival using exogenous molecular and cellular agents to promote regeneration of the vasculature. These are based on stimulation of angiogenesis by extracellular and cellular components. This review article carries out a systematic analysis of the most recent scientific literature on the application of stem cells in patients with CLI. The results obtained from the detailed analysis of the recent literature data have confirmed the beneficial role of cell therapy in reducing the rate of major amputations in patients with CLI and improving their quality of life.

## 1. Introduction

Critical limb ischemia (CLI) is an important condition in the general population with a strong social impact [1]; the prevalence of CLI in the population aged 60–90 years is estimated as 1% (0.5–1.2%) [1, 2] with male to female ratio around 3 : 1 and 5–10% of patients with asymptomatic peripheral arterial obstructive disease (PAOD) or claudication will progress to CLI at 5 years from the first diagnosis. Several studies have shown that over 50% of CLI patients do not have any PAOD symptoms 6 months prior to the onset of CLI [3]. The major risk factors for PAOD include smoking, hyperlipidemia, hypertension, and—for development of CLI—diabetes. Diabetic patients are, at least, fivefold more likely to develop CLI than nondiabetic patients.

CLI is the end stage of PAOD and the macrovascular lesions induce a reduction of distal perfusion. Nutrient blood flow to the tissues and microcirculation exchange are severely altered [4].

Strategies to treat CLI and its related symptoms include both pharmacologic therapy and invasive procedures [5]; however, about 25% of patients still progress each year to limb amputations [6]. Pathophysiologically, chronic ischemia exceeds tissue capacity for oxygen diffusion and nutrients from peri-ischemic territories, as well as for endogenous remodeling. Recent therapeutic strategies have focused on restoring this balance in favor of tissue survival using exogenous molecular and cellular agents to promote regeneration of the vasculature: these are based on stimulation of angiogenesis by extracellular and cellular components [7–9]. Several studies have shown that bone marrow-derived endothelial and hematopoietic progenitors may restore tissue vascularization after ischemic events in limbs, retina, and myocardium [10–14]. Dysfunction in the vascular bed in ischemic conditions, attrition of the microvasculature, and the difficulty or impossibility to adapt to the need for increased blood flow are the critical points through which we investigate cellular mediators and tissue-specific chemokines, which facilitate selective recruitment of bone marrow-derived stem and progenitor cells to specific organs and the factors that promote differentiation of the progenitor cells [15, 16]. The different families of chemokines are determined by the numbers and spacing of cysteine residues adjacent to the amino terminus: CC, CXC, CX3C, and XC. The CC chemokines primarily attract mononuclear cells, including monocytes, eosinophils, basophils, dendritic cells, and T lymphocytes. CXC chemokines primarily attract neutrophils (CXCL1–3 and CXCL5–8) or lymphocytes (CXCL4 and CXCL9–16). Peripheral blood monocytes express CCR1, CCR2, CCR3, CCR5, and CXCR4. Evidences show that a large cohort of chemokines affects monocytes/macrophage recruitment and consequently influences arteriogenesis and response of tissues to ischemia.

The principle that characterizes the therapeutic application of stem cells is the restoration of vascular cellularity, the control and the support of the newly formed vessels which must ensure an adequate supply of oxygen in critical ischemic areas. Thus, oxygen tension plays several roles in the expression of different genes such as the vascular endothelial growth factor (VEGF) family and proangiogenic growth factor.

The aim of this study is to perform a systematic analysis of the most recent scientific literature on the application of stem cells in patients with CLI of different etiologies.

## 2. Material and Methods

PubMed, Scopus, and ScienceDirect databases were searched for articles using the terms: Peripheral Arterial Obstructive Disease, Critical Limb Ischemia, Stem Cells Therapy, Angiogenesis and Limb Loss.

Only publications in English were included. Titles and abstracts were screened by 1 author (B. L.) to identify potentially relevant studies. All potentially eligible studies were subsequently evaluated in detail by 1 reviewer (B. L.) through consideration of the full text. Reference lists of retrieved articles were also searched for relevant publications.

Inclusion required clinical trials in which therapy with stem cells in CLI patients was performed. Studies were excluded if not performed in English language, if performed in animals or in vitro, if the cohort was defined by the presence of CLI and an additional confounding disease process (e.g., chronic renal failure or cerebrovascular diseases), or if CLI specific results could not be distinguished from those of a larger population consisting of individuals without CLI. Studies were excluded when the primary focus was carotid artery disease, aortic aneurysmal disease, intracranial vascular disease, inflammatory diseases, cancer, nonvascular diseases, and treatment with chemotherapy.

## 3. Results

**Figure 1:**
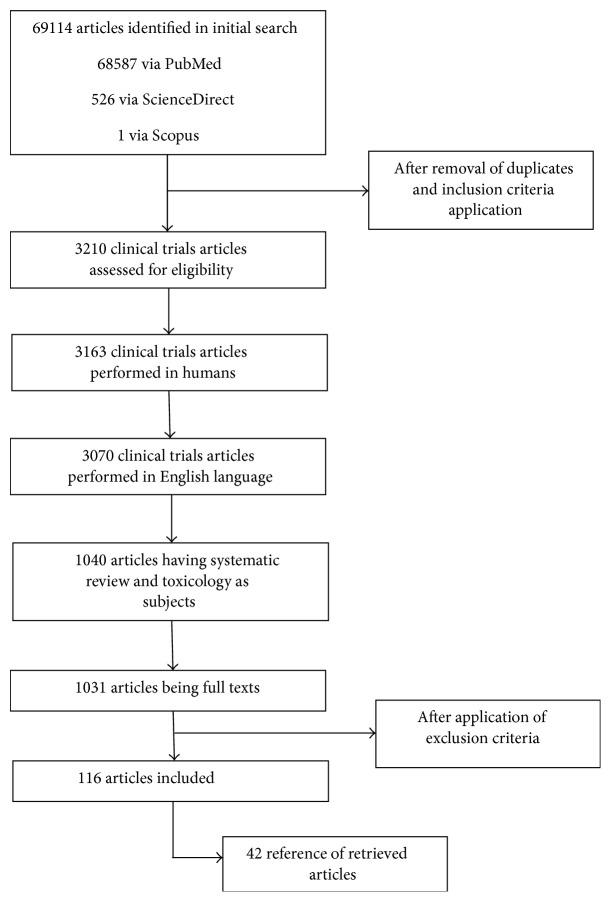
Flow of papers identified from search strategy.

*Study Selection*. The initial database searches yielded 68587 studies from PubMed, 526 from ScienceDirect, and 1 from Scopus in the last 5 years. We evaluated 1031 eligible full text articles (Figure 1).

The biology and physiology of stem cells and their differentiation in vascular cells, the current methods of sampling of stem cells found in literature, the relationship with clinical and adverse effects in treated patients, and the description of the indications to the stem cell therapy in patients with CLI are given below.

### 3.1. Biology of Vascular Stem Cells

Embryonic stem cells (ESCs) have the competency to self-renew indefinitely while maintaining the potential to give rise to all cell types in the human body; the first human cell line was generated in 1998 by Thompson et al. [17]. Many studies were made to clarify the physiology of stem cells, the stage specific embryonic antigens, and the several factors which maintain “stemness” [18–25]. During embryogenesis, the inner cell mass (ICM), the internal cell component of the blastocyst, gives rise to the primitive endoderm and epiblast, which consists of three primary germ layers: ectoderm, mesoderm, and endoderm. Vascular cells including endothelial cells (ECs) and vascular smooth muscle cells (VSMCs) are predominantly descendants of mesodermal cells; however, an ectoderm origin for VSMCs was detected [26, 27]. The differentiation of mesoderm in vascular cells is regulated by important factors in a complex process with a fine regulation: Brachyury, a transcription factor required for posterior mesoderm formation and differentiation and then downregulated when cells undergo specific development into mesoderm-derived tissues, including cardiac muscle, endothelium, and blood cells [28, 29], bone morphogenetic protein (BMP), a member of the transforming growth factor- (TGF-) *β* superfamily [30, 31], MIXL1, a homeobox gene involved in hematopoietic specification [32, 33], Nodal [34, 35], CD31 [34], [36], CD34 [37, 38], Sca-1 [39, 40], N-cadherin [41, 42], platelet-derived growth factor receptor- (PDGFR-) *α* [43, 44], and vascular endothelial growth factor receptor- (VEGFR-) 2 [45, 46].

Blood vessels arise from endothelial precursors through a process known as developmental vasculogenesis [47, 48]: resulting capillaries are small and cannot sufficiently compensate for a large occluded transport artery due to Hagen-Poiseuille law [49, 50]. Arteriogenesis, also called collateral growth, is the transformation of preexistent collateral arterioles into functional collateral arteries: evidences have shown that human bone marrow-derived stromal cells promote arteriogenesis through paracrine mechanisms [51, 52].

Studies showed that ischemia induces plasma elevation of stem and progenitor cell-active cytokines, including soluble kit-ligand (sKitL) and thrombopoietin, progenitor-active cytokines such as granulocyte-macrophage colony-stimulating factor (GM-CSF), and erythropoietin. Thrombopoietin and sKitL may release stromal-derived factor-1 (SDF-1) from platelets accelerating revascularization of the ischemic limbs through mobilization of hemangiocytes [50, 53, 54]. Hemangiocytes induce neovascularization by releasing angiogenic factors and by physically supporting the assembly of endothelial cells. The risk factors due to insufficient collateralization (diabetes, smoking, hyperlipidemia, and advanced age) are the same for a lower number of circulating, monocytic progenitor cells (MPCs) [55–57].

Also immature VSMCs play a central role in blood vessel morphogenesis; they proliferate and migrate and produce extracellular matrix (ECM) components of the blood vessel wall such as collagen, elastin, and proteoglycans. Vascular growth stimuli, such as ischemic injury, in large and small vessels can trigger a process of differentiation of VSMC in which the matrix proteases, known as matrix metalloproteinases (MMPs), may play several roles: MMPs, thus, are not only involved in many vascular [58–72] and nonvascular diseases [73].

### 3.2. Mobilization of Stem Cells

The hematopoietic stem cell (HSC) resides in the bone marrow (BM) but several chemokines and cytokines have been shown to enhance trafficking of HSC into the peripheral blood. This process, known as stem cell mobilization, results in HSC microenvironmental interactions with the critical ligands, receptors and cellular proteases. Peripheral blood progenitor cells (PBPCs) advantages are avoidance of general anesthesia and pain and other adverse effects related to BM collection. Studies have shown that peripheral blood after cytokines stimulation contained a major number of CD34+ cells and T and NK cells than those in BM collection [74].

Granulocyte colony stimulating factor (G-CSF) and GM-CSF represent the two major cytokines used to mobilize the stem cells. These two factors can stimulate the stem cells that are released from the BM niches into peripheral blood. The BM niche is a structured microenvironment composed of supporting cells that anchor, through cell interaction, stem cells and regulates the self-regeneration, proliferation, and release into the circulation. Supporting cells also provide stem cells survivor with chemical signals: neurotransmitters induce membrane type-1 metalloproteinase (MT1-MMP) expression and MMP-2 activity [58–72, 75], which mediate the cleavage of ties (CXCR4, VLA4, VCAM-1, and SCF) holding the stem cells in the BM niche and supporting their blood release.

Several studies have shown that administration of G-CSF and GM-CSF leads to a dose-dependent increase of endothelial progenitor cells (EPCs) in peripheral blood [76–78]. G-CSF promotes not only granulocyte expansion but also reduction of adhesion molecules and disruption of the SDF-1/CXCR4 axis: proteolytic enzymes, neutrophil elastase (NE), and cathepsin G (CG) cleave adhesion molecules as VCAM-1, SDF-1 and CXCR4, and c-kit [79, 80]. CXC chemokine receptor-4 antagonists can mobilize EPCs increasing MMP-9 signaling in the BM [58–72, 81, 82].

GM-CSF, instead, is rarely used because it mobilizes a reduced number of cells compared to G-CSF [83]. Generally, VEGF, fibroblastic growth factors, and stromal cell-derived factors have the ability to recruit EPCs; parathyroid hormone, statins,and other ligands may be used to mobilize stem cells, alone or in combination with G-CSF [84, 85].

Stem cell treatments with BM-derived cells (BMDCs) show safety outcomes but also adverse events related to cell collection and mobilization. Porat et al. postulated that alternatively activated dendritic cells (DCs) can promote the generation of EPC-enriched stem cells within a one-day culture [86].

Another source of stem cells can be satellite cells of skeletal muscle: these cells are one of the well-studied adult tissue-specific stem cells and have served as an excellent model for investigating adult stem cells. Myogenic precursor cells of postnatal muscle are responsible for the repair and regeneration of muscle fibers in adult tissue, either by fusing together and forming new fibers or incorporating themselves into damaged muscle cells and their myonuclei [87]. Satellite cells are mitotically quiescent or slow-cycling, committed to myogenesis, but undifferentiated. Satellite cells are the only source of new myoblasts in the adult tissue but they decrease with the age. In ischemic conditions these cells can be activated and their behavior is similar to those of bone marrow stem cells [88]. Satellite cells are activated by Myf-5 [89], a transcription factor, and CD34 is required for maintaining the quiescent state of myogenic stem cells [88]. After 6 hours from injury, satellite cells are activated and migrate, after disruption of basal lamina, from adjacent myofibers by projecting across tissue bridges initiated from an outpouching process of the satellite cell itself [90, 91]. Currently, the limit of this method is represented by the low number of in vivo studies which show the effectiveness of neoangiogenesis in patients with CLI.

Mesenchymal stem cells (MSCs) are multipotent cells showing adaptability and secretory capacity: thus, they can mediate reparative processes from the through release of soluble molecules, MSC-derived growth factors, and extracellular matrix components with paracrine mechanisms. It is likely that lower secretion of these important factors is the cause of failed tissue reparation. Studies have shown that cells obtained from older patients with multiple risk factors have impaired functions [92, 93]: for this reason, therapeutic success in CLI patients could be increased by using MSCs from young donors.

Therapeutic administration of stem cells does not have to be derived only from bone marrow but also from adipose tissue [93–95] and umbilical cord [96, 97] and other sources and released cytokines are main driving molecules in reparatory processes in CLI patients [98–100] with different results (Table 1).

### 3.3. Intramuscular versus Intra-Arterial Administration of Stem Cells

Intramuscular and intra-arterial injection or a combination of both may be proposed in the treatment of human PAD. The principle of intramuscular injection is the creation of a cell depot with paracrine activity in the ischemic area. Experimental animal studies indicate that BM-derived cells contribute to vascular and muscle regeneration by physically integrating into the tissue and/or by secreting growth factors [101, 102]. The principle of intramuscular injection is the creation of a cell depot with paracrine activity in the ischemic area. Injection of bone marrow mononuclear cells has been reported to promote neovascularization of ischemic tissues effectively. This angiogenic effect may be related to their ability to induce vascular and muscle regeneration by direct de novo vascular and muscle differentiation or paracrine mechanisms through vascular endothelial growth factor secretion. Bone marrow mononuclear cells (BM-MNCs) contained the cell fractions that include EPCs and released various angiogenic factors: incorporation of EPCs in newly formed vessels as well as angiogenesis/arteriogenesis by angiogenic factors released from injected cells likely contributes to the increase in blood. Studies indicate that BMDCs contribute to vascular and muscle regeneration by physically integrating into the tissue and/or by secreting growth factors [103–105].

Intramuscular injection was performed into the gastrocnemius muscle; furthermore, injections were also placed along the occluded native arteries, because the density of preformed collaterals is highest in parallel orientation to the axial arteries: this is the preferred location for collateral growth [106, 107].

The effects of intra-arterial or intra-arterial plus intramuscular cell administration were compared to the effects of intramuscular cell administration. Ankle-brachial index (ABI) and transcutaneous partial pressure of oxygen (TcPO_2_) were found to be significantly improved only after intramuscular or combined therapy and not after intra-arterial cell therapy only [50, 108, 109]. On the other hand, significantly improved pain and pain-free walking distance were detected and there was no difference between the two. Intramuscular cell therapy significantly improved ulcer healing, while this could not be assessed in detail in trials of intra-arterial cell therapy. Pilot studies by the small number of patients reported the improvement of clinical signs and symptoms of intractable patients with CLI by injection of G*-*CSF [110, 111] or intramuscular injection of G-CSF-mobilized peripheral blood mononuclear cells [112–114]. Tateno et al. [115] postulated that the implanted peripheral blood mononuclear cells stimulate ischemic skeletal muscle cells to produce muscle-derived angiogenic factors, thereby promoting neovascularization. In all studies, there were no significant differences in the clinical characteristics between patients treated with intra-arterial or intra-arterial plus intramuscular cells [50, 116, 117].

### 3.4. Adverse Effects in Stem Cells Therapy

Injection of BM-MNCs significantly improved pain-free walking time, rest pain, and tissue oxygen pressure on average 6 months after treatment, whereas injection of peripheral blood mononuclear cells did not exert significant effects [118, 119]. Several studies reported very low mortality rate (<15%) in patients treated with autologous stem cells implantation for CLI [108, 120–122]. These findings suggest that the angiogenic cell therapy using intramuscular implantation of BM-MNCs is valid therapeutic choice and not inferior to the conventional revascularization therapies in patients with CLI. Because of the increased risk and the reduced potential of the treatment, peripheral blood stem cell treatment is less appropriate in the older age [123]. In some studies, deaths have been reported: these were mostly due to acute myocardial infarction, congestive heart failure, and stroke while perforation peritonitis and sepsis have been reported as exceptional [124, 125]. In 2012, Jonsson et al. reported a high incidence of serious adverse events in patients treated with peripheral blood mononuclear cells, causing the investigators to terminate the study [126]. Out of 9 patients, 2 had a myocardial infarction that was believed to be related to the bone marrow stimulation and 1 of the 2 patients died. Another patient had a minor stroke 1 week after stem-cell implantation.

As previously showed, hemodialysis [127], diabetes mellitus [128, 129], and complication with coronary artery disease (CAD) [130–132] are factors that negatively impaired angiogenesis or limb salvage in animal experiments and clinical settings.

### 3.5. Patients in Whom Endovascular and Surgical Revascularisation Are Not Believed to Be Possible

Patients with poor outflow vessels and extensive comorbidities resulting in unacceptable risk of a revascularization procedure, as well as patients who had previously failed revascularization attempts, are not candidate to surgical and/or endovascular procedures [133–136]. This subgroup of patients without revascularization options, known as NO-CLI, is a population with a high rate of limb loss and death.

One primary endpoint for evaluating the outcomes of NO-CLI therapy is major amputation (AMP), which is usually combined with mortality for AMP-free survival (AFS) [137–140]. AFS captures two hard endpoints, mortality and amputation, that are obviously important to patients and clinicians. Despite the fact that the AFS and mortality are the focal points used in the majority of RCTs in the literature for NO-CLI, they necessarily require periodic review and updating.

In the absence of arterial reconstruction options, novel approaches, such as pharmacologic, gene, or stem cell therapy, are proposed; in particular, regenerative medicine has recently emerged as a new speciality that has created great expectations in the scientific community [141]. Improvement of neovascularization is a therapeutic option to rescue tissue from critical ischemia.

## 4. Discussion

The gold-standard treatment of severe PAOD and CLI is surgical or endovascular revascularization. However, up to 30% of patients are not candidate for such interventions, due to excessive operative risk or unfavorable vascular involvement. Despite the progress in medical and surgical therapy of patients with CLI, the prognosis of patients with no option for revascularization remains poor: the amputation rate is high as well as mortality rate (20%) within six months.

During the last two decades, a novel therapeutic strategy has been proposed: the stem cells therapy. BMDCs include autologous BMCs, BMMNCs, and EPCs; peripheral blood-derived cells include PBMNCs, PMNCs, ECFCs, CPCs, and EPC, while other cells mainly include MSCs and ADSCs. In 1997, Asahara et al. discovered that bone marrow-derived circulating cells are able to differentiate into endothelium and promote new vessel growth. These cells, known as EPCs, were able to improve tissue perfusion in myocardial and peripheral ischemia through the stimulation of vasculogenesis [142]. As showed in our review, the studies which have applied the stem cell therapy in NO-CLI patients are very numerous. Despite some failures associated with factors that invalidated the functionality of the different stem cells (i.e., diabetes), the results obtained from the detailed analysis of the recent literature data have confirmed the beneficial role of cell therapy in reducing the rate of major amputations, improving distal perfusion, increasing walking distance, reducing pain, improving ABI and TcPO_2_, and improving overall ischemic symptoms in patients with CLI and their quality of life (Table 2).

Bone marrow aspiration was well tolerated, the most frequent adverse reaction being local pain, responsive to nonsteroidal anti-inflammatory drugs; common adverse event was mild anemia. G-CSF stimulation was generally well tolerated, with prevalently minor side effects, including flu-like symptoms, myalgia, fever, and bone pain. Intramuscular or intra-arterial delivery associated with intramuscular injections of BMMNCs cells had positive results in the majority of clinical studies: the procedure appeared to be generally safe and well tolerated and most adverse reactions were expected given the severe underlying disease and could not be directly attributed to cell therapy. The intramuscular administration seems preferable maybe because cells could hardly reach the target tissue when infused intra-arterially in severely compromised arterial beds.

Thus, bone marrow cells or peripheral blood cells administration? The latter seems to be easier to perform and it might be repeated but it does not appear to be inferior in efficacy: BMMCs seemed to be more effective than mobilized peripheral blood cells in inducing reparative processes because these cells are transiently dysfunctional due to cleavage of the chemokine receptor CXCR4, which is directly involved in stem cell homing. PB-MNCs show comparable or even superior efficacy in comparison to BM-MNCs. In conclusion, BMCs, BM-MNCs, and PB-MNCs are the main cell types used and there is no clear superiority of one cell type over the others. Current literature supports that intramuscular BM cell administration is a relatively safe, feasible, and possibly effective therapy for patients with CLI not susceptible to conventional revascularization.

Based on the recent literature data, treatment-induced improvements are sustainable at 2-3 years: if long-term efficacy becomes definitively established, the stem cell therapy for severe inoperable PAOD will be strongly enhanced. For this reason, multicenter, large-scale and randomized controlled clinical trials may be fundamental and mandatory to prove the safety and efficacy of promoting angiogenesis by the administration of stem cells and for this therapy to become a standard treatment strategy for the patients suffering with CLI.

Another key aspect is that stem cell therapy is an expensive treatment and its cost-effectiveness has not been determined. Thus, a detailed cost-benefit analysis is desirable.

## Figures and Tables

**Table 1:** Comparison of advantages and limitations of different types of stem cells.

Stem cell type	Limitations	Advantages
Embryonic stem cells	Ethical dilemmas, possible immune rejection after implantation, a small number of differentiated cardiomyocytes being generated, leading to teratocarcinomas; genetic instability	Differentiating into cells of all three germ layers
Pluripotent stem cells	Genetic instability, more research needed before using for cardiovascular repair/regeneration	Avoiding ethical concerns
Adult stem cells	Natural regeneration capacity of CSCs being too limited, acquisition and isolation difficulties, more research needed	Avoiding ethical concerns, lower risk of immune rejection
Mesenchymal stem cells	More research needed	Allowing for allogeneic grafting without the use of immunosuppressive agents, self-renewal, proliferating, and differentiating, promoting growth of adjacent cells, less susceptible to mutations, easy to collect
Hematopoietic stem cells	High maintenance, low frequencies, unknown signaling pathways	Proliferating and migrating to injury site in response to physiological/pathological stimuli, capable of myogenesis and angiogenesis
Endothelial progenitor cells	Extremely low numbers in peripheral blood and bone marrow making ex vivo expansion difficult	Increasing its numbers in response to ischemia/cytokine stimuli and migrating to injury site and differentiating into new myocytes

**Table 2:** Clinical trials using stem cells for treatment of critical limb ischemia.

Authors	Type of cells	Clinical outcomes
Nizankowski et al. [143]	BMCs	Improvement of symptoms (pain, cold sensation), increase in ABI and TcPO_2_, new collateral vessels
Napoli et al. [144]	BMCs	Increase in ABI and walking distance, ulcer healing, reduction of amputation rates
Procházka et al. [145]	BMCs	Improvement in toe pressure, TBI, LDI, and TcPO_2_
Matoba et al. [146]	BMMNCs	Long-term improvement in pain scale, ulcer size, and walking distance, reduced amputation rates
Amann et al. [8]	BMMNCs	Limb salvage, increase in ABI and TcPO_2_
Kawamura et al. [147]	PBMNCs with GcSF	Reduced amputations, mostly in nondiabetic nondialysis patients
Huang et al. [148]	PBMNCs versus BMMNCs	PBMNC administration: higher overall efficacy, improvement in ABI, skin temperature, rest pain, walking distance, TcPO_2_, ulcers, and amputation rates (both treatments)
Tateishi-Yuyama et al. [149]	Bone marrow MNCs	Increased ABPI and TcPO_2_ pressure, decreased rest pain
Motukuru et al. [150]	Bone marrow MNCs	Increased ABPI and improvement in ulcer healing
Lara-Hernandez et al. [108]	Peripheral blood CD34+ CD133+ cells after G-CSF mobilization	Increased ABPI and improvement in ulcer healing
Benetti et al. [151]	Human fetal-derived stem cells	
Burt et al. [152]	EPCs (CD34/CD133)	Improvement in amputation-free survival, exercise capacity, pain relief, collateral formation, perfusion, and QoL
MESENDO (II) Clinicaltrials.gov # NCT00721006 [153]	Stem cell mixture	Completed; pending publication
Lasala et al. [154]	BM-MNC EPC + BM-MSC	↑ABI, ↑angiogenesis (MRA), ↑AFS, ↑TcPO_2_, ↑WH, ↑WT, ↓pain
Dash et al. [155]	BM-MSC	↑angiogenesis (biopsy), ↑WH, ↑WD, ↓pain
NCT01257776 [156]	Adipose-MSC	ABI, AFS, DSA improved
NCT01216865 [156]	Cord-MSC	ABI, AFS, pain, WT, WH improved

## Abbreviations

ABI: Ankle-brachial index
ADSCs: Adipose tissue-derived stem cells
AFS: AMP-free survival
AMP: Amputation
BM: Bone marrow
BM-MNCs: Bone marrow mononuclear cells
BMDCs: BM-derived cells
BMP: Bone morphogenetic protein
CAD: Coronary artery disease
CG: Cathepsin G
CLI: Critical limb ischemia
CPCs: Circulating progenitor cells
DCs: Dendritic cells
ECM: Extracellular matrix
ECs: Endothelial cells
ECFCs: Endothelial colony forming cells
EPCs: Endothelial progenitor cells
ESCs: Embryonic stem cells
G-CSF: Granulocyte colony stimulating factor
GM-CSF: Granulocyte-macrophage colony-stimulating factor
HSC: Hematopoietic stem cell
ICM: Inner cell mass
MMPs: Matrix metalloproteinases
MPCs: Monocytic progenitor cells
MSCs: Mesenchymal stem cells
MT1-MMP: Membrane type 1 matrix metalloproteinase
NE: Neutrophil elastase (NE)
NO-CLI: Patients with CLI and without revascularization options
PAOD: Peripheral arterial obstructive disease
PBMNCs: Peripheral blood mononuclear cells
PBPCs: Peripheral blood progenitor cells
PDGFR-*α*: Platelet-derived growth factor receptor-*α*
PMNCs: Peripheral mononuclear Cells
SDF-1: Stromal-derived factor-1
sKitL: Soluble kit-ligand
TcPO_2_: Partial pressure of oxygen
TGF-*β*: Transforming growth factor-*β*
VCAM-1: Vascular cell adhesion protein-1
VEGF: Vascular endothelial growth factor
VEGFR-2: Vascular endothelial growth factor receptor-2
VSMCs: Vascular smooth muscle cells.
